# The Phenomena of Death

**Published:** 1849-01-01

**Authors:** 


ART. V.—*On Death.* In the "*Cyclopædia of Anatomy and Physiology.*" Edited by R. B. TODD, ESQ. M.D. F.R.S. &c. &c.

"LIFE is a terrible reality, and death a tremendous sacrifice—*vere tremendum est mortis sacramentum!* As the hours of health fly over their heads, some look upon their existence as the so-called *El dorado* of Sir Walter Raleigh; others recoil from its darkness, as it were from the murky lair of an evil spirit; and others again wander like ghosts, *silentes umbræ,* along the slippery shores of life, frightened at the length of their own thin shadows; while, not a few, enviable mortals, thoughtlessly commit themselves to the stream, and, laughing gaily, glide along till they approach, and at last rush headlong over, the yawning cataract, lost for ever within the vortex of the foaming abyss beneath.

Death is everywhere,—in the palace, the hospital, the mansion, and the cot,—floating upon the waves of the wide Atlantic, buried beneath the shifting sands of the African desert, perched at the top of the highest peak of the Andes, struggling among the jungles that entangle the course of the Hoogley, climbing the giant Steppes of great Tartary, squatted at the gates of Constantinople, travelling across the sunny plains of Italy, and moored alongside the muddy banks of the metropolitan Thames. It grins upon us in the cholera or plague, smiles bitterly as it snatches away the victim of phthisis in the bloom of youth, dashes us down with a stroke of palsy, or strangles us at midnight with the rupture of a bloodvessel on the lungs. It is here, there, and everywhere,—with the old and the young, the man in the prime of life, and the maiden in the flower of her age, the child in the nurse's arms, the sailor on the last plank in the midst of the storm, and the soldier leading the forlorn hope. All is death,—for the living die as time flies, and the same hour that is giving birth to one is taking away the breath from another. And the end is hidden from us all,—certain as it is, we know not where, nor how, nor when it will be,—perchance by the roadside, or in a stranger's house, on our own bed, or in the foul wards of an hospital, or in the cheerless dormitory of a union workhouse. And why not? For we live in times of so much vicissitude, both public and private, that the thought of our last hour may well indeed give a momentary pang of uneasiness to us all. Look at that mattress of straw, upon which that cadaverous wretch is breathing his last, with his glazing globe fixed on the bare white-washed wall before him—a finger is writing there, 'One moment more, and thou shalt see God!'

The medical profession exercises its functions in those solemn hours, when time and eternity touch each other. The decay of life is the burden of its vocation. The seven ages of man, so finely represented by the poet, are the dramatic mirror in which is reflected the climacteric periods of the older physicians, or the three great epochs of youth, manhood, and age, so accurately specified in the words of science, by modern physiologists. Death is the term of each particular epoch; for childhood dies when youth begins, and the end of youth is but the birth of manhood, while old age closes the scene when manhood has finished its career. Actually, as well as figuratively, each dies or quits the stage, almost without leaving a trace of its existence behind it. Who remembers his life in his mother's lap? And what has the fire and animation of the curly-headed boy to do with the slow perceptions of the bald slip-shod pantaloon? They are manifestly different beings,—their identity remains, but their similarity is gone,—they are different animals, answering the call of the same name. Love, joy, peace—faith, hope, charity,—understanding, knowledge, and wisdom—are the respective attributes of adolescence, maturity, and decrepitude—of the aged Simeon, King David, and the child Samuel.

The ordinary duration of life has undergone little or no change from the Mosaic period, in which, as at the present day, it varied from three-score and ten to eighty years. The Greek physicians and philosophers divided this term into several periods, by a multiplication of the figures, three, seven, and nine; and Plato was, by the Alexandrine sophists, supposed to have attained to the number of perfection, because he died at nine times nine, or eighty-one. The grand climacterics are forty-two and sixty-three; and, however fanciful such calculations may appear, it is nevertheless practically correct that many drop or totter at these two periods, and that those who surmount forty-two, without the manifestation of any fixed disease, usually proceed in safety (accidents apart) as far as sixty-three. At these periods, two opposite changes take place:—on the one hand, an extraordinary renovation of power displays itself, such as the recovery of hearing, eyesight, teeth, hair, strength, and a renewing of exhausted marrow; while, on the other, instead of a renovation of the powers, at the period before us, we sometimes perceive as sudden and extraordinary decline. We behold a man, apparently in good health, without any perceptible cause, abruptly sinking into a general decay.* Sir H. Halford, to whom we are indebted for an excellent essay on this obscure subject, has emphatically denominated it, *Climacteric Disease.* And where the climacteric temperament is lurking, a very trivial excitement proves sufficient to rouse it into action. An act of intemperance, where intemperance was not habitual, may be the first apparent cause of it. A fall, which did not appear of consequence at the moment, and which would not have been, at any other time, has sometimes jarred the frame into this disordered action. A marriage, contracted late in life, has also afforded the first occasion to this change.† In this state, as in extreme old age, the least external cause is sufficient to arrest one of the three functions indispensable to life, and death immediately arrives, as the last term of destruction of the functions and organs. But few persons die at that end, which is the result of age alone. Of a million of individuals, but a very few attain to it; the others die at all periods of life, by accident or disease; and this great destruction of individuals, by causes apparently accidental, seems to enter into the views of nature, as certainly as the precautions she has taken to ensure the reproduction of the species.‡

"Accidental death, in contradistinction to natural death, is a subject particularly worthy of attention. Nothing is more certain than death;

* Mason's Good's Study of Medicine—Climacteric Decay,

† Essays by Sir H. Halford, President, Royal College of Physicians.

‡ Majendie's Physiology, by Milligan, Edinburgh. 1829. Haller estimates the average probability of human life, and deduces the conclusion, that only one in fifteen thousand ever reaches his hundredth year.

nothing is, at times, more uncertain than its reality: and numerous instances are recorded, of persons prematurely buried, or actually at the verge of the grave, before it was discovered that life still remained, and even of some, who were resuscitated by the knife of the anatomist. Pliny, who devotes an entire chapter to this subject, entitled, "de hisis qui elati revixerunt," among other instances gives that of the Roman consul, Avicula, who, being supposed dead, was conveyed to his funeral pile, where he was re-animated by the flames, and loudly called for succour; but before he could be saved, he was enveloped by the fire and suffocated. Bruhier, a French physician, who wrote on the uncertainty of the signs of death in 1742, relates an instance of a young woman upon whose supposed corpse an anatomical examination was about to be made, when the first stroke of the scalpel revealed the truth: she recovered, and lived many years afterwards. The case related by Philippe Peu is somewhat similar. He proceeded to perform the Cæsarean section upon a woman who had to all appearances died undelivered, when the first incision betrayed the awful fallacy under which he acted. A remarkable instance of resuscitation after apparent death occurred in France, in the neighbourhood of Douai, in the year 1745, and is related by Rigaudeaux, to whom the case was confided. There is scarcely a dissecting room that has not some traditional story handed down, of subjects restored to life after being deposited within its walls. Many of these are mere inventions to catch the greedy ear of curiosity; but some of them are, we fear, too well founded to admit of much doubt. To this class belong the circumstances related by Louis, the celebrated French writer on medical jurisprudence. A patient who was supposed to have died in the Hôpital Salpêtrière, was removed to the dissecting room. Next morning Louis was informed that moans had been heard in the theatre; and on proceeding thither he found, to his horror, that the supposed corpse had revived during the night, and had actually died in the struggles to disengage herself from the winding sheet in which she was enveloped. This was evident from the distorted attitude in which the body was found. Allowing for much of fiction with which such a subject must ever be mixed, there is still sufficient evidence to warrant a diligent examination of the means of discriminating between real and apparent death: indeed, the horror with which we contemplate a mistake of the living for the dead should excite us in the pursuit of knowledge by which an event so repugnant to our feelings may be avoided." *

Dying is not the same as going to sleep. For as we sink into slumber, there is a pleasing confusion of the senses which brings before the

* The above paragraph is copied from a very able article by Dr. Beatty, of Dublin, on *Persons found Dead*, in the Encyclopædia of Practical Medicine by Forbes, Tweedie, and Conolly, p. 316, vol. iii. London. 1834.

fading memory a strange commixture of times, persons, things, and places, till we are lost in the deep unconsciousness of repose. Aroused for an instant, just as we are dropping off, we are made aware of a singular intermixture of thoughts blending together past and recent affairs in a not unpleasant, though in a most grotesque, fantastic grouping. The memory, chiefly at fault, is rendered as it were fragmental, halt, and blind ; while the imagination, let loose, runs riot against the better understanding, and sports its fancies in numerous irrational combinations of thoughts, ideas, and living pictures of the soul. Not so in the hour of death. They who die with their heads sound and undisturbed by the workings of a mortal malady, are wonderfully luminous and collected to the last. Perhaps, they are never more alive than when they are dying. Death lights up the soul with supernatural splendour, and lends a torch that illumines the reason with a clear diffusive flame that goes not out as the shadows of the grave close over its burning, vivid, lambent fire. It is not sleep—nay, by the rood, death is not sleep, but only the departure of that living thing, the soul, as it wings its way from off the earth, and takes its flight across the darksome, dread, profound unknown. We have conversed with the dying at the very jaws of death, and heard them give their reasons for the future and the past with a precision and an energy which proved that, however much the mortal carcase was dissolving into nought, the spirit, or the inner man, was more than ever in a conscious and self-existent state of being. It is this supernatural energy in the articles of death, that often deceives the unpractised and inexperienced bystanders, and induces them to believe that the patient is beginning to improve, instead of being at that very moment on the eve of his departure. Weeping friends and relatives, strangers to a scene like this, do not observe the pinched nose, the filmy eye, the long expiring breath, the cold, pulseless, prostrate hand, the supine attitude in bed, and the ghastly sunken features ;—so prepossessed are all their fondest desires with the vain hope, that he who speaks so intelligibly cannot possibly be at the point of death. But so it is : in a few moments more that form has ceased to breathe, and he who had just spoken the thrilling words of life, now lies stretched out at length, a chilly, voiceless lump of clay.

In an age like this, when the acmé of life is a title of honour or a heavy purse, the visions that haunt the chamber of the dead, the dying, and the sick, are unanimously banished to the distant and reprobate regions of superstition, enthusiasm and folly. The old wife's tale and the child's ghost-story dare not compete with the sober reason of the times, or if, by chance, they gain a willing listener, it is one not worthy of credit,—the idle sailor in the forecastle of the ship during a dead calm at midnight, or the peasant who mutters a mysterious legend concerning the gable end of yonder mansion scarcely discernible in the twilight. We live in a crowd, too busy ever to think, and too much pressed upon ever to be alone. The throng passes along the streets—and men, and horses, and carriages, and noble personages, and troops in military array, glitter in the sunshine, and make the long vista of mansions resound with trumpets, and the noise of business, and the constant hum of secular affairs. It is a grand sight, and the heart leaps with eagerness at the animated spectacle. But step aside, and enter beneath this lofty portal, pass along the spacious passage hanging on pillars, and proceed upstairs. Another flight places you above the shining drawing room, with its mirrors, and chandeliers, and gilded furniture, and rich draperies, and leads you to—*the Chamber of Death!* It is awfully silent—the world is shut out, and attendants, with noiseless footsteps, tread lightly across the velvet carpet, and appear and disappear behind those curtains concealing one of no mean note groaning in his last agony. His end is close at hand—wait a little, for it will not be long. Listen!—"Methought," said a hollow guttural voice—"methought I was young again, and there stood beside me my mother chiding me for the past.—Ah, death, thou art hard upon me!—a little more breath—one moment more!—my favourite child is not yet provided for, and my will is unexecuted!" Hark, again. "Methought a voice said to me, To-morrow at noon, and I will be with thee! Who's there? this is the hour—one moment more—not yet!"——a long rattling sound ensued, one last long drawn convulsive respiration, a sob,—and it was finished! A grave personage, in flowing robes, issued from behind the curtains, and approaching us said, in an audible whisper: "It is passing strange, but certainly some aged figure appeared at the foot of the bed, and with him two other forms of more supernal shape, that hovered awhile and withdrew as he spake. As I stand here, I saw them!"—said the venerable lady, bending her grey eye calmly on ours. She was frenzied for the time, and we led her into an adjoining apartment, and withdrew. On returning to the streets, the sun was in the heavens, and the proud day, attended with the pleasures of the world, was all too wanton and too full of gauds to give us audience.*

Scenes such as these are not unfrequent to the medical attendant; and if they do not exactly produce in him a religious tone of mind, they at least tend to make him reflect on the vanity of the world and the futility of its twice-told tale.

For some time we kept a particular record of the mysterious sayings, such as we could gather them, of persons on their death-beds, or of those who were concerned about them; and we put down everything

* "The sun is in the heavens, &c."—*King John.*

most implicitly, without allowing the shadow of a doubt to cross our minds. They formed a curious catalogue of strange imaginings, showing how unsettled or dislocated the intellect becomes in moments of terror or grief. It is unphilosophical to discard these notices with levity and contempt, or to place them aside as accidents unworthy of our attention and consideration. For are they not the operations of the mind? and is not the mind, in all its operations, the peculiar subject of our inquiries? Disturbed conditions, indeed, they are; and, for the time being, not merely disturbed, but diseased; so that, in this sense alone, they are only so much the more interesting, as being the subject-matter of morbid phenomena. For our own part, we own, that visions and hobgoblins always fix our attention the more closely, because we detect in them certain traces of that lofty aspiration after things supernatural, future, and sublime, which, when directed by the rule of faith, become the groundwork of everything holy, great, and good, along the barren track of our mortal pilgrimage. The ideal of a world to come, so frequently abused and so universally entertained, is the invisible bond that links together the most practical of our virtues—namely, prudence, justice, fortitude, and temperance.

Life passes away, and individuals fall off one after another, as Homer says, like the leaves of the trees in autumn, or like the ceaseless succession of waves that break along the sea shore. It is an old similitude renewed in each generation of the world.

On a bleak heath, partly covered with furze, and flanked on either side by a thick wood, lay, in the grey of the morning, the figure of a man half naked, with his pallid cheek in a pool of blood upon the ground. It was a battle-field, covered with the ruins of yesterday's conflict, and torn up with round shot and the serried tramp of manœuvring squadrons. The sun was peeping above the sharp outline of the horizon, while the wretched being, whom we have just noticed, awoke from the long swoon of death, and twice essayed to rise, but twice fell down again with his face upon the clotted gore that bespattered the grass around. "Dying—dying—dying!" he scarcely murmured, as he fixed his fading and unwinking look on the glorious orb—"dying, and never a priest to shrive me for my sins, nor Matty to make my bed for me!—dying, unhousled, unappointed, unaneled!" A film passed over his waning eyeball, and his spirit fled to Him who gave it. The wind whistled merrily across the plain, the gay clouds laughed in the morning mist, and nature sang with joy for the coming day. St. Lawrence Justinian, at his death, when he saw his friends stand weeping around him, bade them dry up their tears, for that, if they wished to remain beside him, they must rejoice rather than mourn, and hail the bright opening of the everlasting doors. Aloysius Gonzago,

Peter of Alcantara, the celebrated St. Theresa of Spain, and many other ecstatics besides, have wept for joy as soon as they foresaw the long-prayed-for day of their departure close at hand. Dr. Heberden relates, that he received a long letter from a phrenzied patient, correctly written in the lucid interval, or lighting up, that shortly preceded his death; and Sir H. Halford, in his elegant essays already referred to, mentions a young gentleman of family, who awoke from a fatal delirium, and, during the brief moments of recollected reason and mental integrity that were granted him, calmly ascertained the nature of his disorder, prudently discharged several obligations on his purse, deliberately set his house in order, and then died. The dictator Sylla, in a burst of passion, in consequence of his hopes being frustrated respecting the restoration of the Capitol, broke a bloodvessel, and, according to the emphatic expression of Valerius Maximus, vomited forth his soul and his life-blood together. Crassus, the eminent Roman orator, fell a victim, apparently, to his love of eloquence, for he died of a sudden pleurisy a few days after having made an animated address to the senate. Pomponius Atticus, Cicero's intimate friend and literary *confrère*, resolutely starved himself to death in his seventy-seventh year, and died with his faculties alive within him almost to the last. One of Pliny's acquaintances did the same, and expired with the word *decrevi* sternly fixed upon his lips. Socrates smiled and conversed over his last draught of aconite, hellebore, hemlock, or henbane; and Hannibal joked about the tedious death of an old man, as he poisoned himself with the inexplicable *annulus*, that has foiled the acumen of the ablest critics in each succeeding age. The Macedonian, after having passed the Granicus, the Issus, and the Arbelus, and touched upon the very confines of Hindostan, sighed for further conquest, impatient to engrave the signet of his name on the remotest sands of earth. In vain did he seek to hide his chagrin beneath the embroidery of his purple pavilion, when he perceived a limit set to his mortal greatness; disappointed, he arose and returned to die at Babylon, the reluctant victim of his own unrivalled power and ambition. His majesty, George IV., commanding to be apprised of the nature of his complaint, patiently expected the fatal hour of his demise; the famous chemist, Sir H. Davy, kept a journal of his daily symptoms, watching the progress of his own decay; and the great natural philosopher, Dr. Wollaston, pronounced judgment on himself, accurately describing the course of the pathological changes that certainly would, as they actually did, terminate his existence. Such persons as these survive death, and their bodies seem to be but the subordinate agents of their loftier wills and understandings.

In these instances, we have attempted to describe the condition of the mind at the brink of death, when the brain dies last; but sometimes, and perhaps more frequently, the brain dies first, long before the other viscera are defunct. In these cases, the mind goes out prematurely, and the patient sinks unconscious, lost to himself and all around him. Bichât, in his physiological researches, *Sur la Vie et sur la Mort,* has admirably described the difference of the commencement of death in the different organs. If the heart suddenly stops, respiration stops also, and the brain becomes unconscious at once. If the brain and spinal cord, upon which respiration depends, are stunned, severed, or compressed, death is instantaneous. If the lungs are suffocated, the brain and spinal cord are stupified, and the heart ceases to act. It is said, that when death does not begin by the heart, the action of this usually continues at least one beat after respiration. Here, the mind dies at the same time with the brain. Decease is easy and rapid, and the person is dead before he can be made aware of his peril. The slow agonizing deaths arise from the slow loss of power of the brain over the lungs, or of the lungs over the brain, by effusion, &c. Old people die as if they were falling asleep, and phthisical patients frequently die ecstatic. The senses may remain for some time after apparent death has happened; and moribund patients, actually speechless and seemingly unconscious, will, when a question is put to them, reply by signs, or the movement of a lip or of a finger. Perhaps they are suffering when we do not know it. Parts of the body may die before the whole is dead, and such dead parts may be separated by absorption, and life go on as before, independent of the loss. Such separation, however, especially if it be extensive, is seldom effected without danger to the entire system, and is generally accompanied with delirium or disturbance of the mental faculties. In fevers, the mind is obscured, and the sufferer dies frantic or comatose. Diseases of the liver cause melancholy, those of the stomach produce anxiety and fear, renal affections render the patient apathetic, while disorders of the lungs or of the bones make him irritable, fretful, and capricious. Drunkards sink haunted with hideous phantoms, gluttons lie oppressed with the nightmare, profligates droop through a distaste of life, and misers are hunted to death by the ghost of their last shilling. The envious pine away with the sallow taint of a jaundiced eye, and the slothful lazily slumber away in the midst of obesity, gout, and the stone. Such people are the same in death as they have been in life; and if their mind survive their bodies, it is only for the purpose of giving vent to their spleen by still harping on their habitual string of thoughts. They do not live in the hour of death because they had already ceased to live in the daytime of life. They are tormented before their time. Dreams of death precede a fatal apoplexy, and singing sweetly in the frenzy of fever is a sure sign of dying.* An intense desire of life is the deepest source of misery there is, for the resolution not to die is of no avail in the last hour. Yet many are well aware of their approaching end, and some (even children) calculate the day and hour of its occurrence to a nicety; whether the prediction verify the event, or the event the prediction, we cannot tell.† In the sixth book of his Epidemics, and at the tenth section, Hippocrates mentions the case of a house-keeper of a Greek gentleman, who, from her own internal sensations, recognised the precise nature of her malady; ‡ and John Hunter, in his Surgical Lectures, edited by Mr. Palmer, says, "We are sometimes so affected as to feel within ourselves that we shall not live, for the living powers having become weak, the nerves communicate this intelligence to the brain, and the mind is thus made acquainted with the state of the body."§

It is needless to describe the features of death, for they are too well known almost to every one; and the Hippocratic countenance, fresh from the easel of a master, has been touched with so vivid a brush, that, after the lapse of more than two thousand years, the hand of time, which blemishes most things else, has only served to heighten and enhance the colouring. The stillness of the repose of the dead has been the theme of all ages. Its "*fair last look*," as Byron calls it, has attracted the gaze of the poet, the philosopher, the moralist, and the divine. Children mistake it for sleep, and infidels have been daunted by what they scornfully style the "*eternal sleep of death*." When the eye sees what it never saw, the heart feels what it never felt:—*it is mine to-day, to-morrow it will be yours!* When the ruffle of life and the anguish of pain have passed away, the features return to their wonted expression—sometimes they smile, and early friends sadly discover, in the corpse of the old man, the placid face of the boy when life was young and all was hope and glee. The chill and changeless brow, the closed untwinkling eyelid, the marble

* "Oh, vanity of sickness! fierce extremes
In their continuance will not feel themselves.
Death having prey'd upon the outward parts,
Leaves them; invisible his siege is now
Against the mind; the which he pricks and wounds
With many legions of strange phantasies;
Which in their throng and press to that last hold
Confound themselves. '*Tis strange that death should sing!*"
K. JOHN.

† Elliotson's Human Physiology. Fifth Edition, pp. 1043—1046.

‡ Œuvres Complètes d'Hippocrate. Par E. Litré. Tome cinquième, pp. 348, 349. Paris, 1846.

§ Palmer's Hunter, vol. i. p. 268. Longman, 1835.

lip, and the motionless hand, are in striking contrast with the fitful fever of all around us, and the toil and trouble, and the hurly-burly of our daily existence.* Time flies, life wastes, the lamp burns, and the oil diminishes.

The great question, so often agitated, both by lay and professional persons, concerning the propriety of communicating to a patient the fact of his approaching end, has generally been left an open one, to be answered according to the exigencies of the moment and the character of the individual to whom intelligence so awful is to be imparted. Upon the whole, perhaps, it is answered in the negative, and certainly as a medical dogma or axiom, nothing definitive has ever been decided upon it. Judging from the conduct of the most experienced practitioners in conjunction with the patient's "*best friends,*" we should be induced to conclude, that the most prudent course to be pursued at this trying moment, is not only to put the best face upon the matter that circumstances will permit, but, what is still more to the point, to take the greatest possible pains to make the dying man believe that he is sure to get well again, and that his recovery is not far off. At least, such has been the result of our private experience during many years practice in the metropolis, where the highest medical authorities seem to agree in judgment with the most enlightened lay decisions respecting the solution of this very difficult and momentous problem. There is only one occasion when such cautious appearances are unceremoniously laid aside, and the naked truth is summarily brought before the sick man's sight, like the fatal warrant, as it were, of his death, which he is called upon to sign, seal, and execute, in the legal shape of a last will and testament, so essential to the future well-being and personal comfort of his executors, assigns, widow, children, creditors, and anxious acquaintances. On all other occasions, save this, it is deemed expedient to banish the truth from the chamber of death, and by means of cheerful looks, false phrases, trivial amusements, and passing diversions, to hoodwink the sufferer's eyes lest he should see the end of the number of his days, and, likewise, by means of drowsy syrups to smoothe the pillow with factitious sleep, and thus court the oblivion which many still

* Every one knows Lord Byron's magnificent and deeply pathetic lines on "Death" in the *Giaour*. Those of Macbeth are not less touching:—

"Duncan is in his grave;
After life's fitful fever, he sleeps well;
Treason has done his worst; nor steel, nor poison,
Malice domestic, foreign levy, nothing can touch him more."

"There shall be no remembrance of the wise no more than of the fool for ever, and the times to come shall cover all things with oblivion: the learned dieth in like manner as the unlearned."—Ecclesiastes, ii. 16.

suppose to be the ultimate desideratum of a happy death-bed. As if it were a real happiness to pass from this world without knowing it; or as if it were more honest and benevolent to deceive a dying man in a manner which no one would have dared to play off against him in his season of health and strength! We own that we should very seriously resent such misbehaviour on the part of any medical friend in whom we had placed our confidence at the time of our mortal sickness, and were we by any unexpected turn of events afterwards to recover, we should feel that we had lost all confidence—not so much in his professional skill, as in his manifest want of medical courage or tact, in failing to communicate what we hold to be a piece of intelligence of the last importance to our spiritual welfare.

The rules to be observed at the time of any considerable illness appear to us so explicit and precise, that we can only wonder how they could ever have been overlooked, and the present round of vague deceptions substituted in their place, except upon the supposition of a mutual understanding between the medical man and his patient, not to introduce any needless cause of disquiet or alarm. It is, however, the candid opinion of some of the ablest practitioners, that mournful intelligence of any kind, such, for instance, as telling a patient he will surely die, is an effectual antidote to recovery, and only ensures the catastrophe which it is his obvious duty to postpone or avert. There is truth in this remark, and no doubt the greatest discretion is to be used in making such startling disclosures; besides which, no medical man can be certain that his prediction, or prognosis, will prove true, for many have died who ought to have recovered and many recovered who ought to have died. No one can control events. Moreover, the communication may be ill-timed, or injudiciously made, or abruptly spoken; all these inconveniences are to be allowed for, although, when allowed for, they do not weigh any more against such a proceeding as this, than they do against the propriety of performing any other business in the world. Want of judgment and inexperience will make anything miscarry.* But, we believe it will be found true, that the communicating to a patient the fact of his being near his end, has a most soothing effect on his mind and spirits, and becomes, paradoxical as it may sound, a very powerful means, indirectly, of promoting his recovery. To him the world has suddenly come to a close—his career has been

* An old lady, who was attended by her physician and apothecary, seriously requested them to inform her whether she was likely to die. After a deliberate consultation, they resolved to comply with her request, and they told her they thought she was near her end. "Very well," replied the sagacious old dame, "then I must dismiss you, because, if you think so, it is evident that you are not the persons to get me well again!" and she accordingly discharged them both. The writer's father was one of the medical attendants.

run — the battle lost and won — his temporal fears are removed — his expectations are directed to a new object—he has nothing to do but to settle his worldly and eternal accounts, and to die. A soothing sleep follows upon this announcement, as is the case with condemned criminals, and more especially in one who is conscious of having done the best he could for the cure of soul and body. Independent of all this, there are certain signs of death never to be mistaken or overlooked; and when these are present, the truth ought to be told. This is much better done at the beginning of the sickness than afterwards, when the strength of the fever, and the quality of the remedies, may render the person absolutely unfit for so great a work. It is the duty of all who surround the sick bed to give timely notice if the distemper be dangerous, and not to flatter with the hopes of life when there is little or no ground for hope. The best use should be made of the time that remains. Debts ought to be discharged, wills executed, obligations fulfilled, enemies forgiven, and pardon sought and bestowed, the affairs of this life brought to a close, and those of the life to come earnestly entered upon. It is as much the duty of the medical man as of the divine to see to this.

Interesting as the investigations of modern physiology have been on the subject of death, and enriched as practical medicine has become by its direct observations on this point, it is singular that so little has resulted from their combined operations in consoling or knowing how to manage the patient, or the patient's friends, in the dark hour of our mortal exit. It leaves them in despair, or, at the best, commends them to the suggestions of their own good sense, or the kind words of some casual or intimate friend. It owns itself impotent, and, as far as its professed teaching goes, shows itself careless or indifferent in providing for the needs of the mind or soul when those of the body have no longer any claim to make on the talents of the physician. He utters his last opinion, and prudently retires from the scene before the curtain drops. To what is this palpable failure owing? From whence comes it, that so much learning and skill have proved fruitless in producing a single medical moralist or philanthropist of any note or consideration? Is it that the medical office really does expire before the patient has heaved his last sigh? or that the medical mind is unable to cope with the moral as expertly as it does with the physical conditions of humanity? Medical literature is barren on this topic; and we turn away from the unmeaning speculations in "*the last days of a philosopher*," with as much dissatisfaction or disgust as everybody now does from the dull materialism of the last century. When D'Alembert, the great geometrician, was on his death-bed, a youth, approaching his side, addressed him tenderly, and said—"Monsieur D'Alembert, you have always been my friend—allow me to ask you a single question. After all that you and your friends have written on Christianity, what appears the most certain to you at this moment?" "Ah!" said D'Alembert in return, touched at the generous impulse;—"Ah! certain!—what is certain?" Such were the last words of philosophy, represented by one of its most distinguished partisans, who had evidently laid open a chasm in that young breast, entirely beyond his power ever to close again. For there is something that escapes the much-vaunted ken of science, however acute and penetrating it may be, in the act of dying, when the fashion of the world is passing away, together with the lust of the flesh, the lust of the eye, and the pride of life.

"What were you saying?" asked Death, who waits upon everything: "What is that you were saying?" "We were saying," replied the great World, "that we wish thou wouldst go away, or change." "Go away, I cannot," rejoined Death; "and as to changing, I never change." "Not change!" exclaims the World; "why everything else is on the change; astronomy changes, chemistry changes, philosophy changes; nay, even empires and dynasties are changing; and *thou!* why dost thou not change?" "Because I belong to God," says Death. "Very true," continues the World; "but dost thou not see that we are the masters of the earth? Look, we have a hundred thousand men under arms,—artillery, cavalry, infantry, shells, shot, musketry, lances, and a fleet of twenty sail of the line—to say nothing of marines, blue-jackets, armed steamers, swivels, and flat-bottomed small craft. We have conquered the East, and have a mind to tear thee out of those musty volumes of the history of ages, and throw thee aside like the worn out leaf of a useless note-book." "Good," says Death; "do as you please,—hell and destruction are mine, and the smell of blood is a relish to my craving maw,—you have my consent." "Agreed," responds the exulting World; "here are the credentials of our power—come, now, let us divide the spoil of the inheritance of this terrestrial globe between us!" "O World!" retorts Death, "thou sayest well! Go, do what thou wilt with thy purple, thy crown of diadems, thy throne of state, and the breath of the nostrils of the hundred thousand soldiers that await thy command. Lo! to-morrow thou thyself shalt be mine, buried beneath six feet by two of green turf, or moist clay, or the cold marble of a costly mausoleum; and then I will have a general illumination or a public mourning observed in honour of thy remains, just as the case may be, for I have been at this work ever since the world began; while you, O you—ye men of this generation—ye are but of yesterday, and to-morrow ye are not!" *

* Our readers cannot be otherwise than much gratified with the perusal of the article on "Death," in Dr. Todd's "Cyclopædia of Anatomy and Physiology." Everything that appears with the sanction of this able and accomplished physiologist, or proceeds from his own pen, has a marked genius stamped upon it.

